# Opportunities for Subnational Malaria Elimination in High-Burden Countries

**DOI:** 10.4269/ajtmh.20-1342

**Published:** 2020-10-27

**Authors:** Kim A. Lindblade, S. Patrick Kachur

**Affiliations:** 1 Malaria Elimination Unit, Global Malaria Programme, World Health Organization, Geneva, Switzerland;; 2 Heilbrunn Department of Population and Family Health, Columbia University Mailman School of Public Health, New York, New York

In this issue, researchers from the Kenya Medical Research Institute (KEMRI) and their global collaborators report on four related studies conducted in an area of Kenya’s western highlands where malaria transmission has historically been low and unstable. The team tracked episodes of confirmed malaria illness through enhanced passive case detection over up to 10 years in two communities with a total population of nearly 8,000. They previously documented the probable interruption of local malaria transmission in 2007 and 2008 following blanket coverage with indoor residual spraying (IRS) of insecticide and introduction of artemisinin combination treatment as first-line antimalarial therapy.^1^ In this issue, they identify the unstable malaria hotspots that emerged as transmission was reestablished,^2^ following a shift in malaria control policy that discontinued IRS of insecticide in the area, and report on the impact of a subsequent insecticide-treated net (ITN) mass campaign.^3^ The team also documents serological correlates of individual protection from malaria illness^4^ and population-level markers of recent and past exposure^5^ to *Plasmodium falciparum* parasites. Collectively, the long collaboration between researchers and public health implementers at these sites provides an important perspective from which to consider how targeting subnational areas for malaria elimination can contribute to reducing the malaria burden.

Recently, there has been increasing appreciation of, and response to, the heterogeneity in malaria transmission at subnational levels.^6^ Rather than taking a “one-size-fits-all” approach to malaria interventions, national malaria programs increasingly are expanding the use of evidence to stratify their districts by levels and characteristics of malaria transmission, to define appropriate goals for different epidemiological strata, and to design and implement the optimal mix of interventions for each stratum. Like Kenya, many malaria-endemic countries, even some with a high overall national burden, include discrete areas where malaria persists only at very low levels. Although these areas contribute little to the overall burden of malaria in a particular country, targeting them for elimination could, perhaps paradoxically, help the country reach its goals for reducing malaria morbidity and mortality more quickly.

Working toward and attaining elimination in certain subnational areas could generate substantial enthusiasm and motivation for national reductions in the malaria burden. At a global level, we have already seen evidence of this phenomenon: elimination of malaria in Sri Lanka in 2016 inspired dozens of other countries to attempt the same feat, leading to recent elimination of malaria in 10 countries that were endemic in 2015, and achieving one of the milestones for 2020 established in the Global Technical Strategy for Malaria 2016–2030.^7,8^ Importantly, the continued, committed support of politicians and administrators for investment in malaria control and elimination is built through demonstrations of success, and elimination of the disease, even at the subnational level, is unquestionably a victory. Friendly competition between regions helps increase and maintain political will, whereas implementation of elimination activities in selected subnational areas strengthens the national program by creating a record of progress and learning that will be relevant to other areas of the country.

Subnational initiatives to interrupt malaria transmission followed by validations of elimination were used effectively in the Chinese national malaria elimination program that is now approaching WHO certification.^9,10^ Kenya itself has included an objective in its national malaria strategic plan 2019–2023 to establish systems for malaria elimination in targeted counties by 2023.^11^ The experiences described in the KEMRI reports have shown that it is possible to interrupt malaria transmission in the western highlands of Kenya, but sustaining this achievement will require a program that is fit-for-purpose for elimination. Such a well-documented setting, with decades of research collaboration, can become a crucible for consolidating and testing malaria program capabilities that will eventually be used nationwide. Key among these is a surveillance system that can capture and report individual cases in time to investigate and take action. When as few as three or four cases a week are confirmed in an area, it should be feasible to investigate and classify each individual case, reliably identify local transmission foci, and initiate an appropriate response. The numbers of cases reported from health facilities in the study areas in western Kenya in recent years may already be low enough to undertake individual case investigations. Transmission foci, even unstable hotspots, could be further characterized through active and reactive case detection and then targeted for further intervention. Reorienting vector control efforts in this setting to areas with the highest levels of receptivity for malaria could improve the cost-effectiveness of vector control. In addition, supplemental ITN replacement or reactive IRS of insecticide could augment responses to imported or introduced cases.

Progress toward achieving the global malaria targets for morbidity and mortality in 2020 has stalled. Without galvanizing a renewal of effort, increased funding, and new tools, achieving the 2025 and 2030 targets is also in jeopardy. The COVID-19 pandemic is likely to make the malaria situation even worse.^12^ Achieving subnational elimination in low-transmission settings of high-burden countries could provide a needed jolt to accelerate the global effort to defeat malaria.
